# Determinants of motor, language, cognitive, and global developmental delay in children with complicated severe acute malnutrition at the time of discharge: An observational study from Central India

**DOI:** 10.1371/journal.pone.0233949

**Published:** 2020-06-01

**Authors:** Navneet Khandelwal, Jagdish Mandliya, Kamna Nigam, Vandana Patil, Aditya Mathur, Ashish Pathak

**Affiliations:** 1 Department of Pediatrics, AIIMS, Bhopal, India; 2 Department of Pediatrics, R. D. Gardi Medical College, Ujjain, India; 3 Department of Women and Children’s Health, International Maternal and Child Health Unit, Uppsala University, Uppsala, Sweden; 4 Global Health—Health Systems and Policy, Department of Public Health Sciences, Karolinska Institutet, Stockholm, Sweden; 5 International Centre for Health Research, Ujjain Charitable Trust Hospital and Research Centre, Ujjain, India; IRCCS materno infantile Burlo Garofolo, ITALY

## Abstract

**Background:**

Undernutrition leads to impaired psychosocial and cognitive development. This study explored the developmental status of children with complicated severe acute malnutrition (SAM) and correlated it with various risk factors for SAM.

**Methods and findings:**

We recruited 100 children with SAM and no other associated significant health issues during the recovery phase of treatment using the Bayley Scales of Infant and Toddler Development III prior to discharge from the nutritional rehabilitation unit in R D Gardi Medical College, Ujjain, Central India. We also assessed composite developmental scores, developmental age equivalents, and average differences in developmental age. Risk factors for developmental delay were identified in children with complicated SAM. The results revealed that 75%, 75%, and 63% of children with SAM exhibited delay in motor (mean score: 78.22), language (mean score: 83.97), and cognitive (mean score: 78.06) domains, respectively. A total of 63% children exhibited delay by an average of 4–7 months in the total developmental age. The proportion of children with delay in motor, language, and cognitive domains was determined. An increased risk of global developmental delay was observedin children with a low birth weight (adjusted odds ratio [aOR]: 18.06, 95%CI: 2.08–156.56; *P* = 0.009), having working mothers (aOR: 17.54, 95%CI: 3.02–102.59; *P* = 0.001), weight-for-age less than three standard deviations (aOR: 6.09, 95%CI: 1.08–34.10; *P* = 0.04), and presence of severe anemia (aOR: 16.34, 95%CI: 2.94–90.73; *P* = 0.001).

**Conclusions:**

The results indicated that children with SAM exhibit developmental delay across all domains. Identifying multiple modifiable risk factors for developmental delay in children with SAM will be helpful in devising early interventional strategies in low-middle income countries; however, the exact timing of such interventions should be investigated.

## Introduction

Undernutrition and severe acute malnutrition (SAM)are global pediatric public health problems [1, 2].Owing to acute undernutrition globally, 52 million under-5 children experience wasting (approximately 7.7%) and 17 million under-5 children experience SAM [1]. Children with weight-for-height below three standard deviations, mid-upper arm circumference below 11.5 cm, and bilateral pedal edema or visible wasting are considered to have SAM [3].

In 2013, undernutrition in children resulted in 1.3 million deaths and 120 million disability-adjusted life years (DALYS) in this population [1, 2]. According to National Family Health Survey-4 data for the state of Madhya Pradesh (MP), India, where this study was undertaken, 43% of under-5 children years are underweight, 42% are stunted, 25.5% are wasted, and 9.2% are severely wasted [4].These rates are much higher than the national averages of these nutritional indicators. SAM significantly contributes to mortality in under-5 children, as children with SAM have a nine-fold increased risk of mortality [5]. In addition, SAM leads to growth retardation and results in impaired psychosocial and cognitive development [6, 7]. Studies evaluating the mental development of children with SAM in early childhood have indicated that these children retain marked cognitive deficits for many years [6–9]. However, with early intervention, higher cognition levels can be achieved in SAM children [10–12]. The policymakers do not recognize that the cognitive effects of SAM can be reversed [10]. In India, no study has used a quantitative developmental scale to assess developmental problems in children with SAM. Certain factors such as age, gender, parental literacy level, income, and occupation, family size, anemia, and birth weight are associated with SAM and child development [13]. The primary objective of this study was to assess motor, language, cognitive, and global developmental outcomes in children with SAM using Bayley Scales of Infant and Toddler Development III (BSID III). Secondary objectives were to determine the number of children with SAM who exhibited developmental delay and to explore the risk factors for developmental delay in these children.

## Methods

### Study population and design

This cross-sectional study was conducted at a nutritional rehabilitation unit (NRU) attached to the Department of Pediatrics, R. D. Gardi Medical College (RDGMC), Ujjain, MP, India from March 2015 to September 2016. Consecutive children who were admitted to the NRU during the study period were eligible to be included in the study. Between 6 and 59 monthsof age SAM was defined as [14]:

- Weight/height or weight/length <-3 *Z* score, using the World Health Organization(WHO) growth Charts; or
- Presence of visible severe wasting; or
- Presence of bipedal edema of nutritional origin; or
- mid- upper arm circumference (MUAC) < 115 mm.

If a child fulfilled any of the above criteria an “appetite test” was performed [14] in which the child was offered a minimum amount of local ready-to-use therapeutic food (LRUTF) prepared from groundnut (25%), milk powder (30%), sugar (30%), and vegetable oil (15%) by weight [15]. The amount of LRTUF was determined by the weight of the child as per Indian Academy of Pediatrics (IAP) guidelines [14], which conform to the composition guideline provided by WHO in 2007 [16]. Only children failing the appetite test were admitted to NRU and were eligible to be included in the study after written informed consent was obtained from the children’s caregiver. Children having cerebral palsy, genetic neurological and neuromuscular conditions significant congenital diseases, and malignant diseases, and HIV positive children were excluded from the study. In the children who were admitted in the NRU, SAM was managed as per the IAP consensus statement and the Government of India guidelines [3, 14]. NRU is a public–private partnership between RDGMC and Government of MP. Children with SAM children are referred from *aganwadi’s* (early childhood care and education centres) from Ujjain district. The Government of MP pays daily wage compensation of up-to 200 Indian rupees for 14 days to the mother/caretaker of child admitted to NRU.

### Clinical evaluation

At admission, a detailed history of factors associated with SAM was obtained through a pre-designed questionnaire after obtaining written informed consent. The questionnaire included demographic factors, nutritional history, feeding patterns, comorbidities associated with SAM and relevant history. After admission, the findings of physical examination and anthropometry were recorded. Children with SAM were classified as underweight, stunted, wasted and stunted and wasted as per the WHO/Government of India criteria [3]. Weight was measured using a digital weighing scale (Accord Electronics, Malad, Mumbai, India) to the nearest 0.1 kg. Head circumference and MUAC were measured using a non-stretchable measuring tape. Length was measured using an infantometer (IndoSurgicalsPvt. Ltd., New Delhi, India) for children below 2 years and a stadiometer (IndoSurgicalsPvt. Ltd., New Delhi, India) for those above 2 years. All anthropometric measurements were supervised by a senior resident or faculty member. All children were clinically evaluated from their admission till discharge thrice daily by a team of junior and senior residents, and twice daily by a consultant professor (JM/AP). The relevant history was reported through parent interview. The birth weight was recorded from birth records directly. Development was assessed before discharge during the recovery phase of SAM when the children were gaining at least 10 g/kg weight per day, had a good appetite, and were playful and interactive. BSID (III) was used for developmental delay assessment [17]. The original scale (US norms) was not changed. The scale was forward translated from English to Hindi by two bilingual paediatricians. A panel consisting of two language experts resolved any discripencies in translation. Back translation was done by an independent language expert to see if original meaning was retained. BSID (III) was administered in a quiet, well-lit, and comfortable room by two trained paediatricians simultaneously in presence of mothers after informing them regarding the significance of identifying developmental delay for relevant interventions. For 12 months and younger and 13 months and older children, the administration time was, respectively, an average of 50 and 90 minutes. Development was assessed in five domains: gross motor, fine motor, expressive language, receptive language and cognition. Raw scores were converted to composite scores, as outlined in the manual [17]. To calculate the language composite-score, an average of the expressive and receptive language scores was obtained, and the motor composite score was calculated by averaging gross and fine motor scores. The average motor, language, and cognitive scores were compared with the scores in the standard score sheet in the manual [17]. For all children, developmental age equivalents were calculated for each of the five domains from raw scores [17]. An average developmental age was calculated from the developmental age equivalents of the five domains. To calculate the average difference in the developmental age, the chronological age of the child was subtracted from the developmental age equivalent. BSID (III) is not validated for Indian children.

### Outcome variable

Children exhibiting mild, moderate, and severe delay in all three domains, namely motor, language and cognitive domains according to the composite score on BSID (III) were classified as exhibiting global developmental delay which was the outcome variable.

### Definitions

For the study, the socioeconomic class was defined according to revised Kuppuswamy’s socioeconomic status scale [18]. Overcrowding was considered to be present if two persons, above 9 years of age, of opposite sexes, not husband and wife, had to sleep in the same room [19]. Low birth weight (LBW) was defined as a birth weight of less than 2500 g (up to and including 2499 g) [20]. Breastfeeding was considered exclusive if the infant has received only breast milk from his/her mother or a wet nurse, or expressed breast milk, and no other liquids or solids. Severe anemia was defined as hemoglobin less than 7gm/dL. Diarrhea was defined as three or more liquid or semi-liquid stools [i.e. able to take the shape of a container] during the past 24 hrs [21].

### Sample size calculation

The sample size was calculated using Stata^**®**^(Version 12.0 Statacorp Texas, USA) for one-sample comparison of the population proportion. To calculate the sample size, a prevalence rate of 2.5% for developmental delay was used based on a community study in south India [22].This study was used to calculate sample size as there was no other study reporting delay in all the developmental domains in SAM.Assuming a 10% confidence interval around the proportion of 2.5%, two-sided alpha of 0.05 and power of 90%, the estimated minimum sample size was 85.

### Statistical analysis

Statistical analysis was done using Stata^**®**^ (Version 12.0 Statacorp Texas, USA). The Shapiro–Wilk test and normal quantile plots were used to determine whether continuous variables were normally distributed. Continuous variables are presented as means and SD, while categorical variables are presented as absolute and percentage values. Two-sample *t* tests and a Mann-Whitney rank-sum test (in the case of nonnormally distributed variables) were used. Pearson Chi-square or Fisher’s exact tests were used to assess differences in proportions.

Pearson Chi-square test was used to test for each independent variables association with the outcome-global developmental delay. Stepwise multivariate logistic regression with backward elimination was used to develop the final model. In bivariate analyses, a *P* value of <0.1 was considered significant for entry into the model. The analysis was started with the full model, then the variable with highest *P* value was removed, and model was revised and refitted with remaining predictors. The procedure was repeated till the *P* value of all the predictor variables was less than 0.1 except age and sex; we designated this as final model. Adjusted odds ratios (aOR) and their respective 95% confidence intervals (CI) were then calculated from the final model and a *P* value of <0.05 was considered significant. We used the Bonferroni-Holm method to correct for multiple testing [23]. For the final model, model discrimination was done using C-statistics-receiver-operating-characteristics (ROC) curve, while model calibration was done using Hosmer-Lemeshaw “goodness-of-fit” test [24–26].

### Ethical approval

The study was approved by the Institutional Ethics Committee of R.D. Gardi Medical College, Ujjain (Approval number-458/2014). A written informed consent was obtained from the parents of the children included in the study.

### Internal validity

During the study, the BSID (III) was done by two trained paediatricians simultaneously. The inter-rater reliability coefficient was excellent (intraclass correlation coefficient (ICC) = 0.85–0.92). High internal validity was achieved by Cronbach’s alpha coefficient for BSID (III) of 0.87. ICC and Cronbach’s alpha coefficient were obtained because BSID (III) is not validated for Indian children.

## Results

### Characteristics of study population

**Fig 1:**
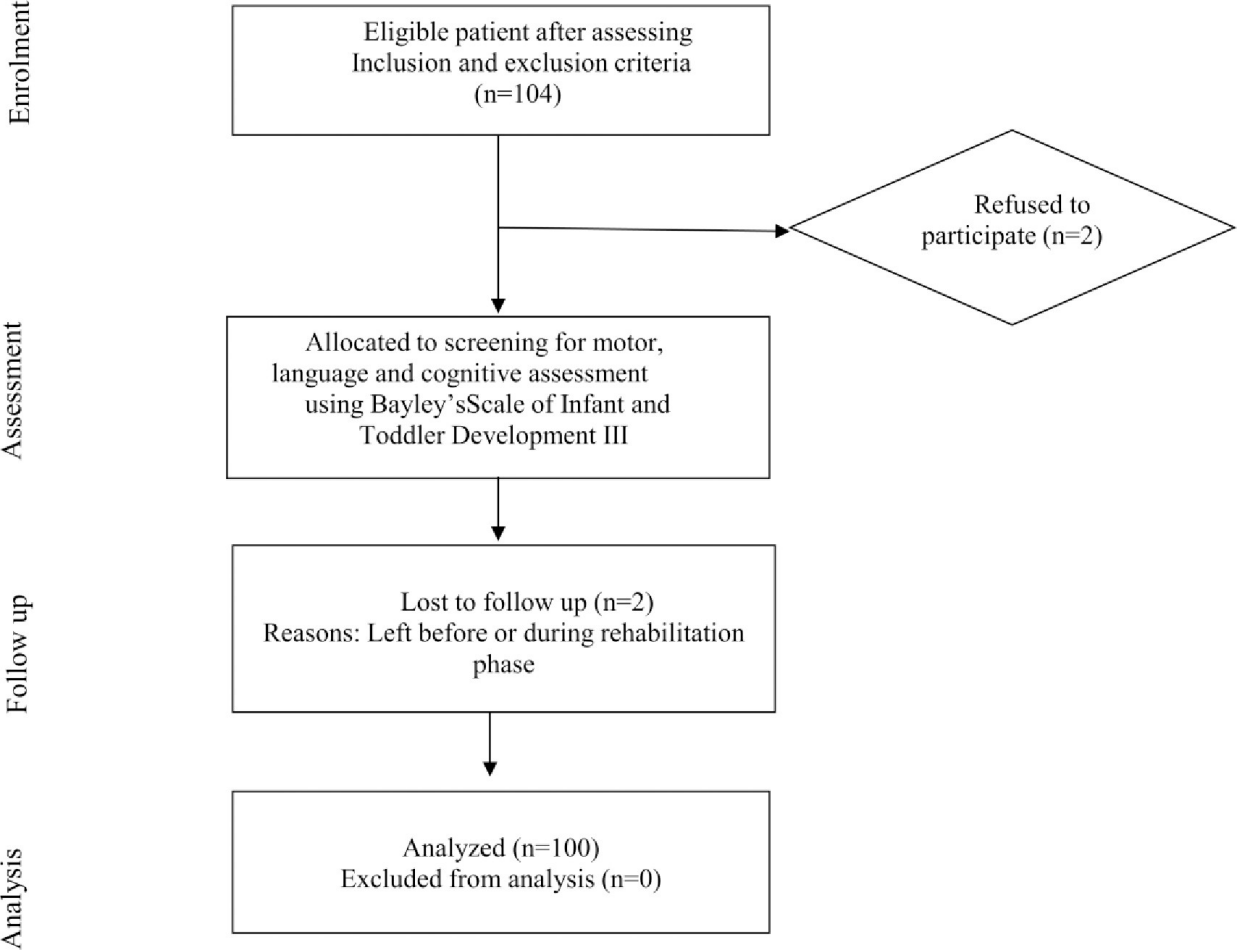
Flow chart of children recruited in the study.

Out of the 104 children available for inclusion, 100 were analyzed. A flow chart of children included in the study is shown in Fig 1.

Admitted children met the following inclusion criteria: weight for height/length <-3 SD (n = 87), MUAC < 11.5 cm (n = 57), visible wasting (n = 18), and bipedal edema (n = 7). All children exhibiting visible wasting (n = 18) fulfilled at least one other inclusion criteria. Of the 100 children with SAM included in the study, 90% were between 6 and 24 months, and 52% were girls with an equal proportion from rural and urban areas. Most (95%) mothers were illiterate or received only primary grade education, whereas 90% fathers received education till primary and middle school. Majority (58%) of mothers were nonworking, and majority (87%) of fathers were unskilled or semiskilled workers. The socioeconomic status according to modified Kuppuswamy classification showed that most (67%) children belonged to the upper-lower class (Table 1). No children belonged to upper and upper middle classes, which are the top two tiers of the socioeconomic status.

**Table 1:** Association of sociodemographic variables with global delay (cognitive, language and motor) in 100 children admitted with severe acute malnutrition in Ujjain, India.

			**Global developmental delay**							
**Independent variable**	**Total n = 100**		**Yes n = 58(58%)**		**No n = 42(42%)**			**MD**	**95% CI**	***P* value**
			Mean (±SD)	Median(IQR)	Mean (±SD)		Median(IQR)			
Age (in months)	20.02(±5.78)		18.34(±5.08)	18(7)	22.33(±5.94)		21(9)	-3.98	-1.79 to 6.18	<0.001
Weight (in Kilograms)	6.15(±1.31)		6.55(±0.83)	6.6(1.11)	5.60(±1.25)		5.38(1.38)	0.94	1.36 to 0.53	<0.001
Height (in centimetres)	71(±5.62)		72.39(±5.26)	73(6.5)	69.07(±5.59)		68.5(9.5)	3.32	5.49 to 1.15	0.003
Percentile weight for age	56.41(±9.63)		61.92(6.57)	62.44(7.73)	48.79(±7.85)		47.74(8.25)	13.12	16.0 to 10.25	<0.001
Percentile height for age	85.72(±5.92)		88.97(±5.04)	89.54(6.31)	81.23(±3.70)		80.94(4.50)	7.74	9.56 to 5.92	<0.001
Percentile weight for height	72.53(±6.78)		74.90(±4.69)	74.82(4.70)	69.26(±7.84)		67.56(6.68)	5.64	8.13 to 3.14	<0.001
**Independent variable**	** **	**Total n = 100**	**Yes n = 58(58%)**			**No n = 42(42%)**		**OR**	**95% CI**	***P* value**
**Socio economic status**	Lower	13(13)	7(54)			6(46)		Reference	Reference	
	Upper-lower	67(67)	38(57)			29(43)		1.21	0.34–3.70	0.849
	Upper	20(20)	13(65)			7(35)		1.59	0.38–6.62	0.523
**Head circumference**	>3SD	75(75)	40(53)			35(47)		Reference	Reference	
	2 to 3SD	20(20)	15(75)			5(25)		2.62	0.86–7.95	0.088
	<-3SD	5(5)	3(60)			2(40)		1.31	0.20–8.31	0.773
**Severe anemia**	No	33(33)	5(15)			28(85)		Reference	Reference	-
**(Hb<7 gm%)**	Yes	67(67)	53(79)			14(21)		21.2	6.9–64.9	<0001
**Hair changes**	No	43(43)	14(33)			29(67)		Reference	Reference	-
	Yes	57(57)	44(77)			13(23)		7.01	2.88–17.05	<0.001
**Skin changes**	No	75(75)	35(47)			40(53)		Reference	Reference	-
	Yes	25(25)	23(92)			2(8)		13.14	2.89–59.75	<0.001
**Wasting**	No	82(82)	42(51)			40(49)		Reference	Reference	-
	Yes	18(18)	16(89)			2(11)		7.6	1.6–35.3	0.009
**Edema**	No	93(93)	54(58)			39(42)		Reference	Reference	-
	Yes	7(7)	4(58)			3(42)		0.96	0.20–4.55	0.962

MD Mean deviation; OR Odds ratios; CI confidence intervals.

Approximately one-third (36%) of the admitted children weighed less than 2.5 kg at birth (LBW). The feeding pattern of majority (78%) showed that they were exclusively breastfed for the first 6 months of age. Complimentary feeds were initiated by 6 months of age only in 45% along with continued breastfeeding. The quantity and frequency of complimentary feeding was ≤3 feeds per day in approximately60% of children with SAM (Table 2).

**Table 2:** Association of birth weight, feeding history and history of comorbidities as risk factors for global developmental delay (cognitive, language and motor) in 100 children of severe acute malnutrition in Ujjain, India.

			Global developmental delay				
Independent variable		Total n = 100	Yes n = 58 (58%^a^)	No n = 42 (42%^b^)	OR	95% CI	*P* value
**Low birth weight**	No	64(64)	24(37)	40(63)	Reference	Reference	-
	Yes	36(36)	34(94)	2(6)	28.3	6.3 to 28.7	<0.001
**Exclusive breastfeeding**	No	22(22)	19(86)	3(14)	Reference	Reference	-
	Yes	78(78)	39(50)	39(50)	6.3	1.7 to 23.1	0.005
**Bottle feeding**	No	17(17)	14(82)	3(18)	Reference	Reference	-
	Yes	83(83)	44(53)	39(47)	0.24	0.06 to 0.90	0.035
**Initiation of CF† (at 6 months)**	No	55(55)	39(71)	16(29)	Reference	Reference	-
	Yes	45(45)	19(42)	26(58)	0.29	0.13 to 0.68	0.004
**≤3 feeds per day**	No	59(59)	43(73)	16(27)	Reference	Reference	-
	Yes	41(41)	15(37)	26(63)	0.21	0.09 to 0.50	<0.001
**H/o diarrhea (in last one month)**	No	58(58)	24(41)	34(59)	Reference	Reference	-
	Yes	42(42)	34(81)	8(19)	6.02	2.37 to 15.27	<0.001
**H/o of pneumonia (in last one month)**	No	86(86)	46(53)	40(47)	Reference	Reference	-
	Yes	14(14)	12(86)	2(14)	5.21	1.10 to 24.72	0.037

%^a^ Column percentage

%^b^ Row percentage, LBW low birth weight

†- CF complimentary feeding; H/o History of; OR Odds ratios; CI confidence intervals.

The examination and anthropometry findings revealed that 87% of patients were underweight, 39% were stunted, and 87% were wasted, and 18% exhibited visible wasting. General examination revealed severe anemia (Hemoglobin less than 7g/dL) in 67% children, hair changes in 56%, skin changes in 25%, and edema in 7% (Table 3).

**Table 3:** Association of anthropometric and general examination findings as risk factor for global developmental delay (cognitive, language and motor domain) in 100 children with severe acute malnutrition in Ujjain, India.

			Global Developmental delay				
Independent variable		Total n = 100	Yes n = 58(58%^a^)	No n = 42(42%^b^)	OR	95% CI	*P* value
**Weight for age**	>2SD	3(3)	1(33)	2(67)	Reference	Reference	-
	2 to 3SD	10(10)	2(20)	8(80)	0.50	0.28 to 8.70	0.634
	< -3SD	87(87)	55(63)	32(37)	3.43	0.29 to 39.42	0.321
**Height for age**	>2SD	24(24)	9(37)	15(63)	Reference	Reference	-
	2 to 3SD	37(37)	21(57)	16(43)	2.18	0.76 to 6.26	0.145
	< -3SD	39(39)	28(72)	11(28)	4.24	1.43 to 12.50	0.009
**Weight for height**	>3SD	13(13)	6(46)	7(54)	Reference	Reference	-
	<-3SD	87(87)	52(60)	35(40)	1.73	0.54 to 5.59	0.357
**Head circumference**	>3SD	75(75)	40(53)	35(47)	Reference	Reference	-
	2 to 3SD	20(20)	15(75)	5(25)	2.62	0.86 to 7.95	0.088
	<-3SD	5(5)	3(60)	2(40)	1.31	0.20 to 8.31	0.773
**Severe anemia (Hb<7/dL)**	No	33(33)	5(15)	28(85)	Reference	Reference	-
	Yes	67(67)	53(79)	14(21)	21.2	6.92 to 64.9	<0.001
**Hair changes**	No	43(43)	14(33)	29(67)	Reference	Reference	-
	Yes	57(57)	44(77)	13(23)	7.01	2.88 to 17.05	<0.001
**Skin changes**	No	75(75)	35(47)	40(53)	Reference	Reference	-
	Yes	25(25)	23(92)	2(8)	13.14	2.89 to 59.75	<0.001
**Wasting**	No	82(82)	42(51)	40(49)	Reference	Reference	-
	Yes	18(18)	16(89)	2(11)	7.61	1.64 to 35.27	0.009
**Edema**	No	93(93)	54(58)	39(42)	Reference	Reference	-
	Yes	7(7)	4(58)	3(42)	0.96	0.20 to 4.54	0.962

%^a^ Column percentage

%^b^ Row percentage, OR Odds ratios; CI confidence intervals

### Assessment of developmental delay in children with SAM

**Fig 2:**
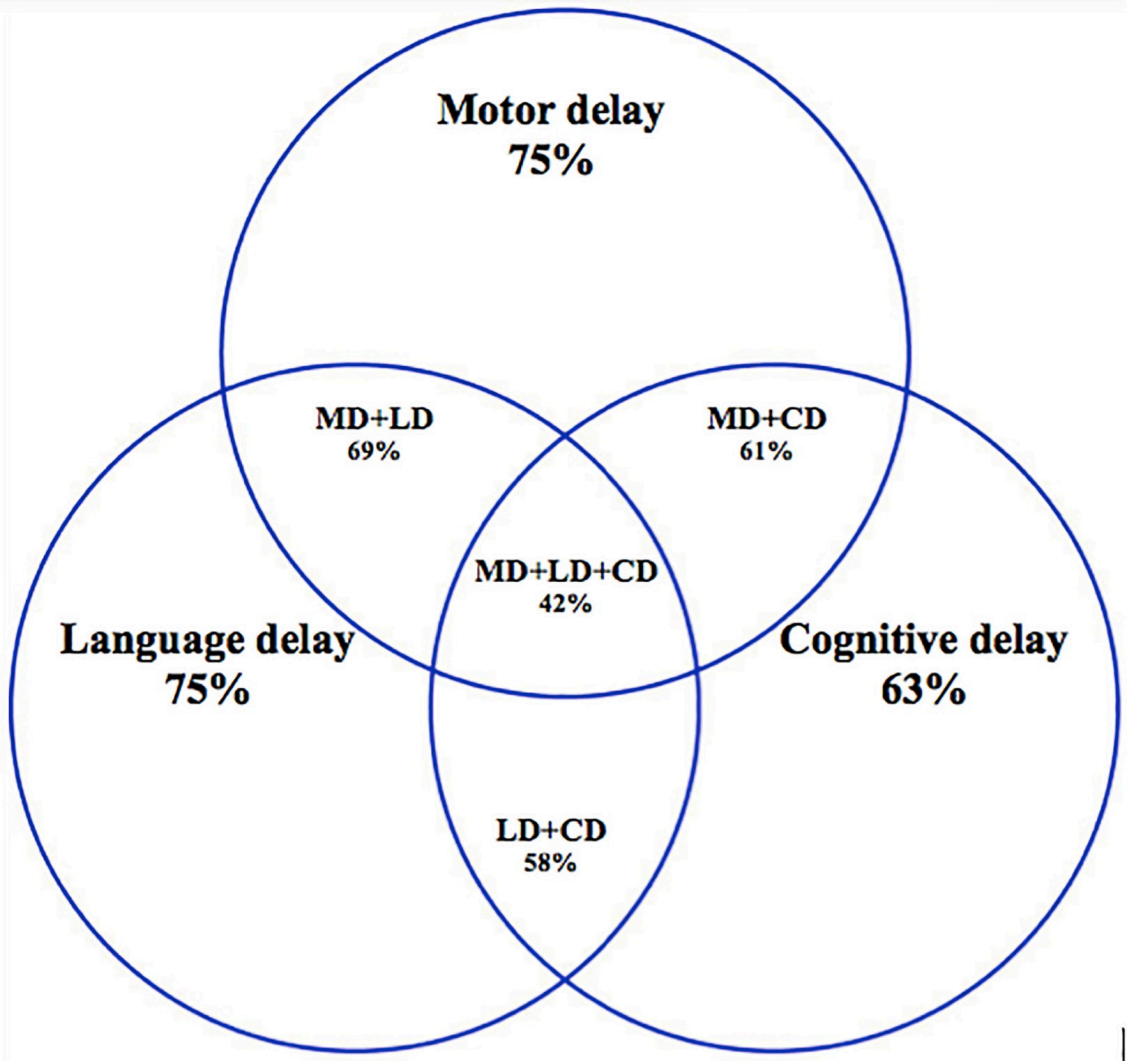
Developmental delay in individual domains and their overlap–motor, language and cognitive domains.

Developmental delay assessment of children with SAM revealed that 75% exhibited delay in motor, 75% in language, and 63% in cognitive domains according to the composite scores (Fig 2). The overlap between the three domains is shown in Fig 2.

A total of 42 children exhibited delay in all three domains. The mean and 95% CI of the motor, language, and cognitive composite scores were 78.06 (95% CI 75.29–80.82), 78.22 (95% CI 75.89–80.54), and 83.97 (95% CI 81.87–86.06), respectively. The average difference in the overall developmental age ranged from +3 to –14 months. Overall developmental delay assessment revealed that 63% children exhibited delay by an average of 4–7 months in the total developmental age. A total of 60% of children exhibited delay by an average of 6–9 months inexpressive language, 49% by an average of 4–6 months in receptive language, 48% by an average of 5–7 months in gross motor, 62% by an average of 3–6 months in fine motor, and 54% by an average of 3–5 months in cognitive domain. Thus, most children exhibited delay in the fine motor domain, but the quantum of delay was the highest in expressive language. No statistically significant difference was observed in the developmental scores of SAM children with or without edema.

### Bivariate and multivariate logistic regression analysis for global developmental delay

Tables 1, 2 and 3 displays bivariate analysis results. Table 4 shows the outcome of multivariate logistic regression outcome of independent risk factors associated with delay in all the domains. An increased risk for global developmental delay was observed with LBW (aOR:18.06), having working mother (aOR:17.54), weight-for-age less than three SD (aOR:6.09) and presence of severe anemia (aOR:16.34).

**Table 4:** Multivariate analyses of variables associated with risk of global developmental delay (cognitive, language and motor domain) in 100 children with severe acute malnutrition in Ujjain, India.

Independent variable	aOR	95% CI	*P* value
**Age (in months)**	0.901	0.785–1.035	0.142
**Sex**	2.056	0.52–8.19	0.306
**LBW present**	18.06	2.08–156.56	0.009
**Working mother**	17.54	3.02–102.59	0.001
**Weight for age less than -3 SD**	6.09	1.08–34.105	0.04
**Severe anemia present (Hb<7 gm%)**	16.34	2.94–90.730	0.001

LBW-low birth weight; aOR adjusted odds ratio; CI confidence intervals

### Model performance

ROC of the final model was 0.9382 showing an excellent model fit. The Hosmer-Lemeshaw test revealed that chi-square was 9.70 (*P* = 0.1378) showing good model calibration.

## Discussion

This is the first study in India that used BSID (III) to assess developmental delay in children with SAM. This study demonstrated that 75%, 75%, and 63% of children with SAM exhibited delay in motor (mean score: 78.22), language (mean score: 83.97), and cognitive (mean score: 78.06) domains, respectively, according to composite scores. A total of 42 children exhibited delay in all three domains. In a bivariate analysis, multiple risk factors were associated with global developmental delay in children with SAM, but in a multivariate analysis, four risk factors—LBW, having working mothers, weight-for-age less than three SD, and presence of severe anemia—were significantly associated with developmental delay.

Malnutrition reduces in the numbers of neurons and their synapses, increases pruning of dendritic connections, and reduces myelination [27], all of which are underlying causes of developmental delay in motor, language, and cognitive domains. Two studies, one from India [28] and another from Malawi [29], have shown progressive reduction in developmental, motor, and mental quotients with increasing severity of SAM. In our study, we found overlapping developmental delay in motor, language, and cognitive domains. In the Malawi study, SAM children with edema (kwashiorkor) had lower developmental scores than those without edema (marasmus) [29]. No statistically significant differences were observed in the developmental outcomes of complicated SAM children with or without edema in the present study, but the results should be cautiously interpreted because in this study, only seven SAM children had edema.

In a study in Tanzania on children with SAM, each unit increase in height-for-age z-score was linearly associated with +0.09, +0.10, and +0.13 higher cognitive, communication, and motor development z-scores, respectively [30]. In the same study, children with SAM who exhibited wasting had −0.63, −0.32, and −0.54 z-score deficits in cognitive, communication, and motor development z scores, respectively, compared to children who did not exhibit wasting [30]. In the present study, both height-for-age and wasting were identified as risk factors for global developmental delay in the bivariate analysis, but they were not statistically significant in the final model.

In the present study, the majority of children with SAM who had developmental delay were aged between 6 and 24 months. Multiple studies have reported that this age group is the most vulnerable for SAM [2, 7, 10, 13, 27]. One of the most important causes of SAM is inappropriate complimentary feeding, with the mother continuing to only breast feeding after 6 months of age [31]. In the present study, children with LBW exhibited more delay in all domains of development than those with a normal birth weight. Multiple studies have reported LBW as a significant risk factor for SAM [13, 28, 30]. In the present study, higher development in all domains was observed in children with SAM having nonworking mothers than in those having working mothers. Other studies from India have outlined maternal factors such as maternal illiteracy [13] and working mothers as risk factors for SAM in children [13]. Severe anemia has been identified as a significant risk factor for global developmental delay in children with SAM. Iron-deficient infants have been shown to have lower developmental test scores than their iron-sufficient peers [32].

The overall poor level of development in children with SAM in this study is of great concern. Studies conducted in Jamaica have demonstrated that the poor nutritional status is linked to educational failure [33]and other studies have demonstrated a link between education and childhood development, intellectual skills, creativity, and wellbeing [34]. Therefore, India should promote increased access to nutritional interventions to boost national development. Because interventions for developmental stimulation in children with SAM are lacking in Indian guidelines, such interventions should be effectively incorporated into Indian guidelines for managing of SAM. WHO guidelines include play therapy and sensory stimulation as methods for developmental stimulation in children with SAM [12].Our study assessed developmental delay in children with SAM after a short duration of inpatient care. Timing of the assessment might not be ideal for developmental assessment in children with complicated SAM. Exact time for assessment remains unknown. Long-term follow-up studies are required because only two such studies have been conducted [35, 36]. Inclusion of child development in the related policies is required to reduce effects of SAM [37]. Following this study, our NRU has implemented an intervention of developmental stimulation for all admitted children with SAM; intervention involves therapists and the mother. The current Indian guidelines of management of SAM do not include a specific developmental stimulation package except for play therapy. Therefore, the results of this study can be used to plan appropriate developmental stimulation for children with SAM in India and other low-middle income countries.

### Limitations

The study has some limitations. BSID (III) used in the present study is not been validated for Indian children. Therefore, we report ICC. High internal validity for the scale was achieved by Cronbach’s alpha coefficient of 0.87. The use of US normed scale in Indian children could potentially skew the scores toward the lower side. The sample size of the study was insufficient to ascertain independent risk factors for each of the domain (motor, language, and cognitive) separately. Certain environmental factors including water, sanitation, and hygiene characteristics have been shown to be associated with SAM, but information on these factors was not was not collected in the present study. Assessment at the time of admission to NRUs would have helped in comparing of the BSID (III) before–after scores but was not performed because of ethical issues.

## Conclusions

Children with SAM exhibit delay across all developmental domains. The quality and quantity of delay can be best assessed through a formal assessment of development by using developmental scales such as BSID (III). Multiple modifiable risk factors for developmental delay in children with SAM were identified in this study. These risk factors will help pediatricians, nutritionists, and child psychologists in resource-poor settings to identify and develop early interventional strategies for children with SAM, ensuring more favorable developmental outcomes in these children.
